# Investigating Quality of Life and Decision Regret in Patients Undergoing Laryngectomy

**DOI:** 10.3390/curroncol33090541

**Published:** 2026-09-09

**Authors:** Dagmawi Lulseged, Jedeiah Dickerson, Lauren Ottenstein, Margie Dixon, Rebecca Pentz, Nicole C. Schmitt

**Affiliations:** 1 Winship Cancer Institute, Emory University School of Medicine, Atlanta, GA 30322, USA; 2 Department of Otolaryngology, Emory University School of Medicine, Atlanta, GA 30308, USA

**Keywords:** total laryngectomy with radiation, salvage laryngectomy, quality of life, swallowing, decision regret

## Abstract

Total laryngectomy is a surgical procedure for advanced laryngeal cancer and can affect speech, swallowing, and breathing. This study compared the quality of life, swallowing function, and decision regret among patients who underwent primary total laryngectomy with adjuvant radiation (Group 1) versus salvage laryngectomy (Group 2). A survey was completed by 25 Group 1 patients and 27 Group 2 patients. Quality of life and swallowing ability were assessed using the University of Washington Quality of Life Questionnaire (UW-QOL) and M.D. Anderson Dysphagia Inventory (MDADI), while decision regret was measured using the Decision Regret Scale. Group 1 patients reported a higher mean total UW-QOL score (74.4) than Group 2 (68.2). Similarly, Group 1 patients demonstrated better swallowing outcomes, with a mean MDADI score of 70.28 compared to 63.90 in Group 2. Decision Regret scores were lower in Group 1 patients (31.4) than in Group 2 (35.37), indicating less regret regarding treatment decisions. However, all these differences did not reach statistical significance. Overall, these findings highlight the importance of fully informing patients of the impact of surgery on quality of life to assist them in their decision-making. Future studies can recruit larger cohorts and investigate the effectiveness of other laryngectomy treatments/procedures.

## Simple Summary

Total laryngectomy is a surgery used to treat advanced cancer of the larynx. While this procedure can be lifesaving, it might affect a person’s ability to speak, swallow, and breathe, making it important to understand how patients feel after treatment. This study compared patients who underwent total laryngectomy as their initial treatment followed by radiation therapy with those who underwent salvage laryngectomy. We examined quality of life, swallowing ability, and regret about treatment decisions. Patients who received surgery as their initial treatment followed by radiation generally reported better quality of life, improved swallowing function, and less regret about their treatment choice. However, these differences were not statistically significant. These findings suggest that both treatment approaches result in similar long-term patient experiences and highlight the importance of providing patients with clear information about potential outcomes when making treatment decisions.

## 1. Introduction

### 1.1. Total Laryngectomy

Total laryngectomy is a surgical intervention for advanced laryngeal cancer which involves the complete removal of the larynx, as well as the creation of a tracheostoma in the neck for breathing [1]. This surgery significantly alters the patient’s anatomy and physiology, affecting functions such as speech, swallowing, and breathing [2,3]. Patients need to utilize an alternative method of communication, such as an electrolarynx, esophageal speech, or tracheoesophageal voice production [2]. In terms of breathing, patients utilize the opening in the neck called a stoma, as compared to breathing through the nose and mouth [1]. Due to these considerable losses of function, this surgery is considered to be a more radical treatment option for advanced head and neck cancer, often used when primary treatments such as chemoradiation are unsuccessful [1].

Previous studies have investigated how these severe functional losses affect patients’ quality of life and can even lead to decision regret following treatment. For example, dysphagia after the procedure is reported to be a persistent burden that decreases comfort, compromises swallowing safety, and limits the overall functioning of patients [4,5]. In addition to swallowing difficulties, patients also experience communication impairments, which are associated with eating difficulties, reduced independence, and social isolation [6]. Other studies have also shown the negative impacts of the procedure on patients’ mood, mental well-being, and daily activities [7]. Furthermore, the need for ongoing rehabilitation and dietary modifications can add to the physical and emotional strain patients experience, reinforcing the long-term impact of treatment.

### 1.2. Total Laryngectomy with Adjuvant Radiation and Salvage Laryngectomy

Total laryngectomy with adjuvant radiation is the use of surgical intervention as the primary treatment, followed by radiation therapy to reduce the risk of disease recurrence [8]. This approach is for patients with advanced-staged tumors or those who have high-risk pathological features, such as multiple lymph nodes [1]. The complications of this therapy include tissue damage and dysphagia, often related to esophageal stenosis.

Salvage laryngectomy, on the other hand, is the surgical removal of the larynx performed after failure of prior radiation for laryngeal cancer [9]. This procedure is utilized when there is recurrent disease that remains localized and surgically resectable [10]. Since the tissues have already been exposed to radiation, they are often fragile, fibrotic, and poorly vascularized, making the surgery technically more challenging [11]. Post-operatively, patients are at increased risk of wound breakdown, infection, and fistula [12,13]. Despite these risks, this procedure can still be curative in selected patients, although the overall prognosis might be less favorable. These key differences are listed in Table 1.

### 1.3. Purpose of the Study

Previous studies have investigated the effects of laryngectomy on patients’ quality of life and decision regret. However, little has been done to characterize these outcomes among patients who have undergone total laryngectomy with adjuvant radiation compared to total salvage laryngectomy. Given the important clinical differences between these two treatments, this study aims to evaluate and compare the quality of life and decision regret outcomes between these groups. By utilizing tools that examine physical and social domains, including communication ability, swallowing function, and emotional well-being, we will provide a comprehensive understanding of how treatment choices shape patients’ lived experiences. The findings may help inform practitioners of good practices to introduce into their care routine for each group of patients. Most importantly, this study provides information that can guide and inform patient-centered preoperative counseling that communicates the potential impacts of surgery on quality of life to assist them in their decision-making.

## 2. Methods

**Figure 1:**
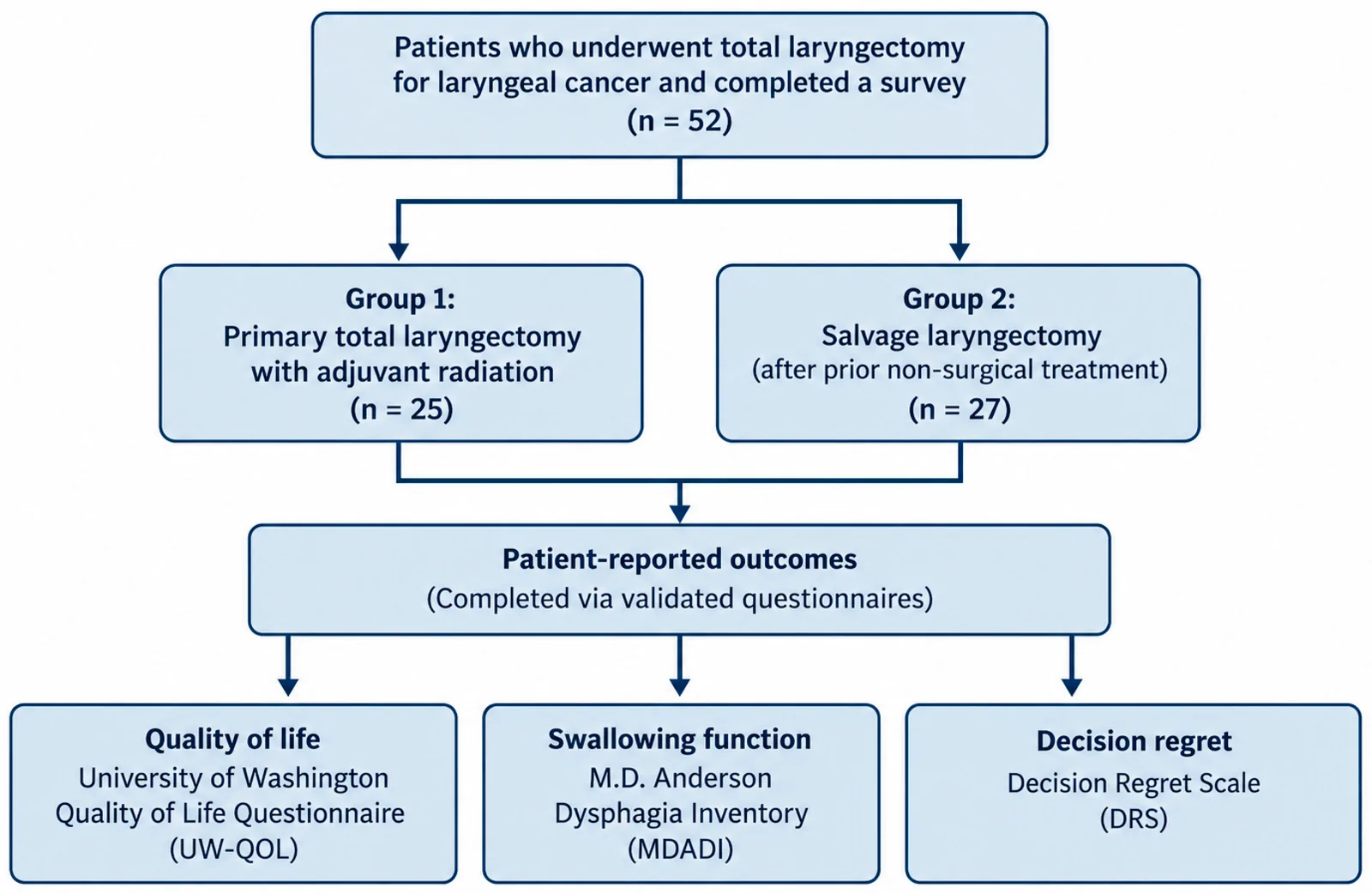
Study design and patient-reported outcome measures.

The head and neck clinical team screened clinic rosters from 2014–2022 to identify patients who had undergone laryngectomy, and asked permission for the ethics team to contact them for study participation. Patients who gave permission were then contacted and provided verbal consent to be interviewed in person or virtually. Patients were divided into two groups: those who had undergone a primary laryngectomy with adjuvant radiotherapy/treatment (Group 1), and those who had undergone a salvage laryngectomy (Group 2) (Figure 1). A total of 52 patients consented to participate in this study (25 in Group 1 and 27 in Group 2). The University of Washington Quality of Life Questionnaire (UW-QOL) was used to assess quality of life. The 12 domains included in the questionnaire are pain, appearance, activity, recreation, mood, swallowing, speech, shoulder, taste, saliva, chewing, and anxiety. Adapted questions from the M.D. Anderson Dysphagia Inventory (MDADI) and the validated Decision Regret Scale were utilized to evaluate the patients’ swallowing ability and their perspectives on their treatment decisions. Survey responses were analyzed according to the instructions for each scale, and statistical analyses were used to determine the results and compare them to normative values and between groups. Statistical analysis was done by Student’s t-test with GraphPad Prism software, version 9. All procedures were approved by the Institutional Review Board at Emory University.

## 3. Results

**Figure 2:**
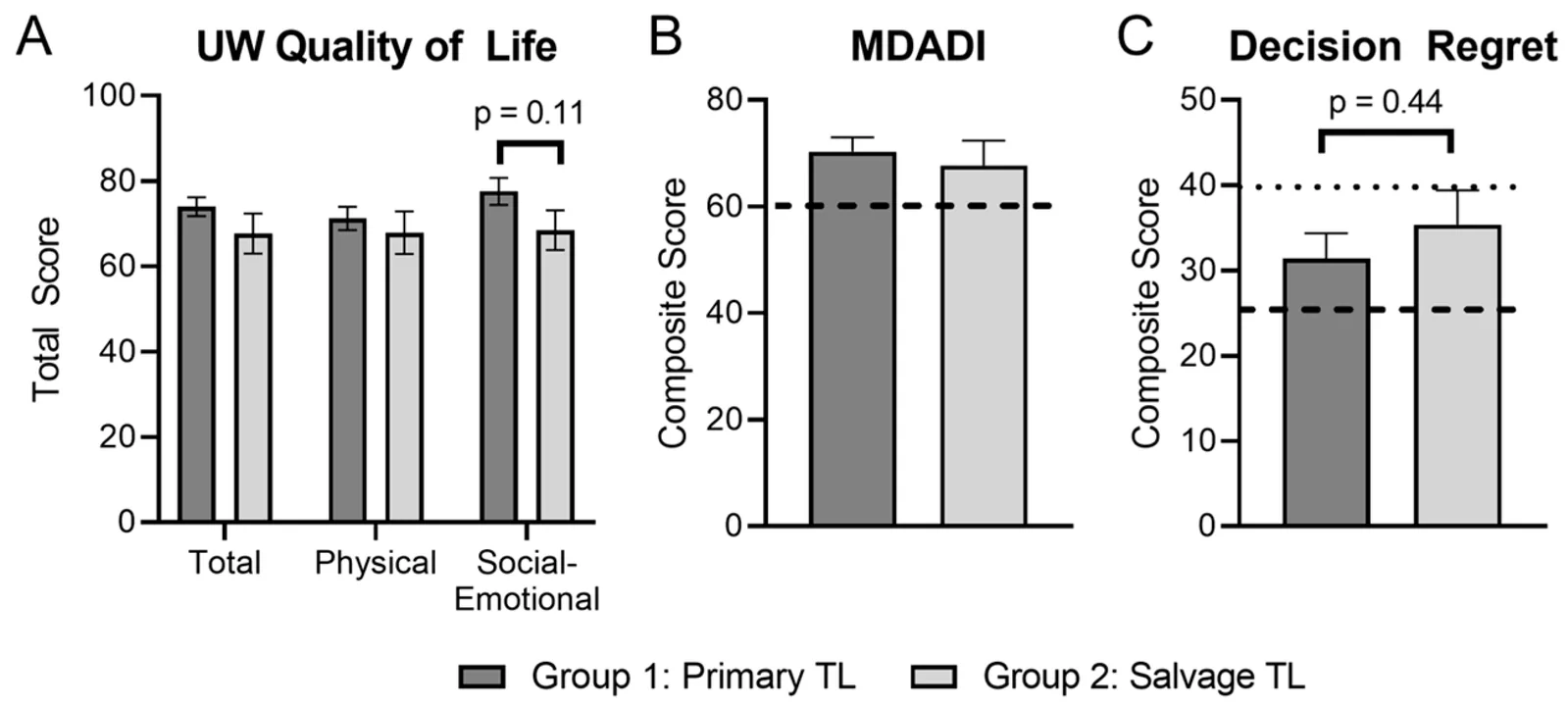
Comparison of quality-of-life outcomes between primary (Group 1) and salvage (Group 2) laryngectomy patients using the University of Washington Quality of Life Questionnaire (UW-QOL), M.D. Anderson Dysphagia Inventory (MDADI), and Decision Regret Scale. (**A**) Mean (+SEM) UW-QOL scores for Group 1 (primary laryngectomy with adjuvant therapy) and Group 2 (salvage laryngectomy). Mean (+SEM) physical and socio-emotional subscale scores for each group. (**B**) Mean (+SEM) MDADI scores for Group 1 and Group 2. Dashed line represents the clinical impairment threshold (60). (**C**) Mean (+SEM) decision regret scores for Group 1 and Group 2. Reference lines indicate thresholds for clinically significant regret (>26, dashed line) and high regret (≥40, dotted line).

From the total participants, 80.8% were white, and 69.3% were male (Table 2). From the UW-QOL analysis, the average quality of life among the 52 patients included was 70.7 on a scale ranging from 0 (worst) to 100 (best). Patients in Group 1 reported a mean total UW-QOL score of 74.4. The mean physical score for these patients was 71.2, whereas their mean socio-emotional score was 77.6 (Figure 2A). Group 2 patients recorded a mean total UW-QOL score of 68.2 (Figure 2A). Their mean physical and socio-emotional scores were 67.9 and 68.5, respectively (Figure 2A). More patients in Group 1 were retired, while more in Group 2 were employed. This comprehensive analysis highlights that patients in Group 1 demonstrated a slightly higher quality of life, though the difference between the groups is statistically insignificant.

For the MDADI analysis, patients in Group 1 demonstrated a higher mean MDADI score (70.28) than those in Group 2 (63.90) (Figure 2B). The 95% confidence interval (CI) for Group 1 (64.6–75.9) remained well above the clinically relevant benchmark of 60, indicating generally preserved function in this cohort [14] (Figure 2B). In contrast, the CI for Group 2 (55.8–72.0) extended below this threshold, suggesting greater variability and a higher likelihood of clinically meaningful impairment among these patients (Figure 2B). However, further analysis showed that there is no statistically significant difference between these groups, though the means may be clinically significant. Overall, these results demonstrate that Group 1 patients exhibit more consistently preserved function, whereas Group 2 patients show greater heterogeneity and an increased risk of clinically significant swallowing impairment.

Analysis of decision regret revealed that both groups experienced levels of regret exceeding the threshold for clinical significance (>26) [15]. However, Group 2 reported a higher mean decision regret score (35.37) compared to Group 1 (31.4), indicating a greater degree of dissatisfaction with treatment decisions (Figure 2C). Similar to the first two parameters, the two groups did not exhibit statistically significant differences, although the values may be clinically significant. While neither group reached the level indicative of complete regret (≥40), the elevated scores in Group 2 suggest a trend toward more pronounced decisional distress relative to Group 1.

## 4. Discussion

Primary total laryngectomy followed by adjuvant radiation and salvage total laryngectomy represent two distinct treatment pathways for patients with advanced laryngeal cancer [16]. The first is performed as the initial treatment for patients with extensive disease, whereas the latter is reserved for patients with recurrent disease, most commonly following radiation or chemoradiation [17]. Because patients undergoing salvage laryngectomy have already received prior treatment, they often present with radiation-induced tissue fibrosis, impaired wound healing, and greater postoperative morbidity [11]. Consequently, salvage laryngectomy has historically been associated with higher rates of complications as well as poorer swallowing and quality of life [17]. Although previous studies have documented these differences, comparatively fewer investigations have directly evaluated long-term patient-reported quality of life, swallowing function, and decision regret between these treatment groups. Therefore, comparing these outcomes is important for understanding the impact of treatment selection and for providing patients with realistic expectations during shared decision-making.

Our study aimed to characterize the quality of life, reported swallow impairment, and decision regret among patients who have undergone total laryngectomy with adjuvant radiation and total salvage laryngectomy. The findings of this study demonstrate statistically insignificant differences in patient-reported outcomes between the two groups, with Group 1 exhibiting slightly more favorable results across quality of life, swallowing function, and decision regret. Taken together, these results suggest that, despite the higher mean UW-QOL score observed in Group 1, this treatment approach was not associated with statistically significant improvements in patient experience or functional preservation compared with Group 2.

The UW-QOL analysis highlights the burden that the disease and suggested treatment impose on patients. Although Group 1 reported higher overall, physical, and socio-emotional scores than Group 2, both groups fell well below normative values reported in non-cancer populations. A prior study on non-cancer patients reported means of 95 and 83 for physical and social-emotional functions, respectively [18], whereas the laryngectomy patients included in our study reported means of 69.55 and 73.1 for Groups 1 and 2, respectively. This highlights the significant burden associated with the disease and its treatment on patient well-being. In addition, our study underscores the persistent impact of head and neck cancer as both groups scored lower than established norms for non-cancer populations.

The MDADI results further reinforce the aforementioned pattern, demonstrating no statistically significant difference between the two groups, though the mean value in Group 1 shows slightly higher swallowing outcomes. The fact that the confidence interval for Group 1 remained entirely above the clinically relevant threshold of 60 suggests that most patients in this group maintain functionally acceptable swallowing ability. In contrast, the wider confidence interval in Group 2, which extends below this threshold, indicates greater heterogeneity and a higher likelihood of clinically significant dysphagia. Given the central role of swallowing in nutrition, social interaction, and overall quality of life, these findings provide an important functional correlation to the broader quality-of-life outcomes.

The Decision Regret Scale has been widely used across oncology to evaluate the quality of shared decision-making and to identify patients who may experience persistent dissatisfaction following treatment [19,20]. The analysis of decision regret adds an additional patient-centered dimension to these results. The fact that neither group reached the threshold for severe regret is notable. However, the elevated regret scores in both groups highlight the importance of thorough pre-treatment counseling, expectation setting, and shared decision-making to better align treatment choices with patient values and outcomes.

The limitations of this study include the relatively small sample size (n = 52) and the lack of longitudinal data. This may limit the generalizability of our findings about quality of life, swallowing function, and decision regret over time. Patient-reported outcomes are also subjective and may be influenced by individual expectations, coping mechanisms, and recall bias. Despite these limitations, the use of validated instruments such as the UW-QOL, MDADI, and Decision Regret strengthens the reliability of the findings. Furthermore, there are fundamental differences between these two groups that we could not adjust for statistically, including differences in employment status and inherent differences in disease/treatment burden at different points in time. For example, patients in Group 1 had more advanced disease at initial diagnosis, requiring laryngectomy; Group 2 faced a longer overall treatment course on average, with higher risk of surgical complications and poor wound healing in the salvage setting. We also did not include a comparison group that responded well to chemoradiation and did not require a laryngectomy; we posit that decisional regret would be lower in that group.

The Decision Regret Scale is written in general terms, so we can only speculate on what factors contributed to regret in laryngectomy patients. Group 2 had multiple decisions to make and a longer overall treatment course. It is also possible that patients in Group 2 were emotionally impacted by ultimately needing a life-altering surgery that they had initially been able to avoid. Future qualitative studies with focused patient interviews would be an interesting way to identify common themes and factors contributing to decision regret.

## 5. Conclusions

In this single-institution cohort of patients undergoing total laryngectomy, patient-reported quality of life, swallowing function, and decision regret were not significantly different between patients treated with primary total laryngectomy plus adjuvant radiation and those undergoing salvage laryngectomy. Although outcomes generally favored the primary-treatment group, both groups experienced meaningful quality-of-life burden and clinically significant decision regret. These findings underscore the importance of patient-centered counseling, realistic expectation setting, and shared decision-making before laryngectomy. Larger, prospective studies with longitudinal assessment are needed to better define how treatment pathway and recovery influence long-term patient-reported outcomes.

## Figures and Tables

**Table 1:** Characteristics of Primary Total Laryngectomy (+adjuvant radiation) vs. Salvage Total Laryngectomy.

Characteristic	Primary Total Laryngectomy + Adjuvant Radiation	Salvage Total Laryngectomy
Indication	Advanced disease with high-risk pathology	Persistent or recurrent disease
Radiation timing	After surgery	Before surgery
Tissue condition	Previously unirradiated	Fibrotic, poorly vascularized
Common complications	Dysphagia, radiation-induced fibrosis	Pharyngocutaneous fistula, wound breakdown, infection
Rehabilitation	Voice restoration, swallowing rehabilitation	More prolonged recovery

**Table 2:** Demographic profile of participants.

	Total Participants (n = 52)	Group 1 (n = 25)	Group 2 (n = 27)
**Age**			
18–50	3 (5.77%)	0 (0)	3 (11%)
51–60	9 (17.31%)	5 (20%)	4 (15%)
61–70	26 (50%)	13 (52%)	13 (48%)
71–80	13 (25%)	6 (24%)	7 (26%)
80+	1 (1.92%)	1 (4%)	0 (0)
**Gender**			
Male	36 (69.23%)	16 (64%)	20 (74%)
Female	16 (30.77%)	9 (36%)	7 (26%)
Other	0 (0)	0 (0)	0 (0)
**Race**			
White	42 (80.77%)	20 (80%)	22 (81.5)
Black or African American	9 (17.31%)	5 (20%)	4 (15%)
Native American/Aboriginal or Alaska	0 (0)	0 (0)	0 (0)
Asian	0 (0)	0 (0)	0 (0)
Native Hawaiian or other Pacific Islander	0 (0)	0 (0)	0 (0)
Other	1 (1.92%)	0 (0)	1 (3.7)
**Hispanic/Latino**			
Yes	1 (1.92%)	0 (0)	1 (3.7%)
No	51 (98.08)	25	26 (96.3%)
**Last grade/School**			
Less than high school	1 (1.92%)	1 (4%)	0 (0)
Some high school	2 (3.85%)	2 (8%)	0 (0)
High school graduate or GED	15 (28.85%)	5 (20%)	10 (37%)
Some college	13 (25%)	7 (28%)	6 (22.2%)
College graduate	10 (19.23%)	6 (24%)	4 (15%)
Some post-graduate	0 (0)	0 (0)	0 (0)
Post-graduate or professional degree	9 (17.31%)	3 (12%)	6 (22.2%)
Other	2 (3.85%)	1 (4%)	1 (3.7%)
**Employment Status**			
Employed full-time	8 (15.38%)	1 (4%)	7 (26%)
Employed part-time	1 (1.92%)	0 (0)	1 (3.7%)
Retired	27 (51.79%)	18 (72%)	9 (33.3)
Not employed	1 (1.92%)	0 (0)	1 (3.7%)
Disabled	15 (28.85%)	6 (24%)	9 (33.3%)
Student	0 (0)	0 (0)	0 (0)
**Income Category**			
<$5000	4 (7.69%)	1 (4%)	3 (11%)
$5000–$19,000	3 (5.77%)	1 (4%)	2 (7.4%)
$20,000–$39,000	6 (11.54%)	1 (4%)	5 (18.5%)
$40,000–$59,000	5 (9.62%)	3 (12%)	2 (7.4%)
$60,000–$79,000	8 (15.38%)	6 (24%)	2 (7.4%)
>$80,000	14 (26.92%)	8 (32%)	6 (22.2%)
Don’t know	3 (5.77%)	2 (8%)	1 (3.7%)
Refused	9 (17.31%)	3 (12%)	6 (22.2%)

## Data Availability

The original contributions presented in this study are included in the article. Further inquiries can be directed to the corresponding authors.
